# Exploration of psychiatric inpatients’ experience of adapting to the COVID-19 pandemic

**DOI:** 10.4102/sajpsychiatry.v31i0.2401

**Published:** 2025-06-30

**Authors:** Muimeleli P. Magwabeni, Isabelle Swanepoel, Marinda Joubert

**Affiliations:** 1 Department of Psychiatry, Faculty of Health Sciences, University of Pretoria, Pretoria, South Africa; 2 Department of Clinical Psychology, Faculty of Health Sciences, University of Pretoria, Pretoria, South Africa

**Keywords:** psychiatric inpatients, experience, adapt, COVID-19 pandemic, mental health care, South Africa

## Abstract

**Background:**

The coronavirus disease 2019 (COVID-19) pandemic has changed life globally and significantly disrupted psychiatric inpatient care, with infection control measures altering therapeutic environments and exacerbating patient distress. Both staff and inpatients had to quickly adapt to new norms while maintaining care in an already vulnerable setting.

**Aim:**

This study aimed to explore psychiatric inpatients’ experience of adapting to the COVID-19 pandemic.

**Setting:**

The study was conducted in Weskoppies Psychiatric Hospital, Tshwane, Gauteng.

**Methods:**

We conducted a qualitative study comprising 15 semi-structured interviews with psychiatric inpatients admitted to Weskoppies Hospital in South Africa during the COVID-19 pandemic, using purposive sampling. Open-ended questions encouraged detailed responses and guided the conversation. Recordings were later transcribed for analysis.

**Results:**

Our study showed that psychiatric inpatients displayed an engagement strategy, rather than a disengagement approach, in adapting to a crisis. They actively tried to control, manage, and change stressful situations by accepting the hospital infrastructure and new COVID-19 rules, seeking social support, and holding on to their faith.

**Conclusion:**

Psychiatric inpatients at Weskoppies Hospital adapted to the COVID-19 pandemic through support from staff, family, hospital systems, and personal coping strategies. These findings highlight the need for holistic, patient-centred care that includes psychosocial and spiritual support during times of crises.

**Contribution:**

This study provided insights into psychiatric inpatients’ experiences and can help mental healthcare practitioners to ensure a more positive experience during rehabilitation and reintegration of psychiatric patients into society.

## Introduction

The coronavirus disease 2019 (COVID-19) pandemic brought about profound changes to healthcare systems worldwide, with psychiatric inpatient settings facing unique challenges. The disruption of routine care limited social interaction and heightened health-related anxieties significantly impacted patients’ experiences and adaptation processes. This study aims to explore the lived experiences of psychiatric inpatients during the COVID-19 pandemic, focusing on how they adapted to the evolving circumstances.

South Africa’s COVID-19 pandemic began on 05 March 2020, with President Ramaphosa declaring a state of disaster and implementing measures like travel restrictions and school closures, and a national lockdown began on 27 March 2020.^1^ This lasted until the national state of disaster was lifted on 05 April 2022.^2^

During this period, millions were infected, with a significant death toll. Economies were destabilised, and healthcare systems were pushed to their limits. However, measures were implemented to minimise the infection rate and reduce the number of lives lost during this pandemic.

While necessary, these interventions had far-reaching consequences for patients, particularly those receiving inpatient psychiatric care. For this vulnerable population, the pandemic altered daily routines, therapeutic environments and interpersonal interactions, potentially impacting their expectations, progress and ability to adapt.

The COVID-19 pandemic significantly impacted psychiatric practice, and contingency plans were developed to manage the unique challenges faced by psychiatric inpatients.^3^ Inpatient psychiatric settings faced challenges in adapting to pandemic-related changes because of their open space design and interpersonal interaction. Patients with behavioural dysregulation struggled with isolation protocols and could not return home to self-quarantine. These limitations increased the risk of transmission and hindered continuity of care. The abrupt routine changes reduced contact with family and staff and increased uncertainty affected patients’ expectations, therapeutic progress and overall adaptation to inpatient treatment during the pandemic.^4^

The COVID-19 pandemic markedly influenced individuals’ well-being, prompting studies examining coping strategies used by mental healthcare practitioners, particularly in response to increased symptoms and acute stress.^3^

Saladino et al.^5^ focused on the effects of the COVID-19 pandemic on the mental health of the general population. The unique context of psychiatric inpatient care presents distinct challenges that require closer examination. As Charles Darwin stated, ‘It is not the strongest of the species that survives, nor the most intelligent that survives. It is the one that is most adaptable to change’.^6^

This study aimed to explore psychiatric inpatients’ experiences of adapting during the COVID-19 pandemic. Although the peak of the COVID-19 pandemic has passed, understanding how psychiatric inpatients experienced and adapted to its challenges remains highly relevant. The insights gained from this study inform mental health practices by highlighting patient resilience, guiding the design of more adaptable inpatient services and strengthening recovery-focused care. These findings may help mental health professionals better support psychiatric inpatients during times of crisis and beyond, ensuring care remains both safe and person centred. Mental health professionals have a crucial role in fostering supportive, adaptable inpatient environments that promote meaningful rehabilitation and successful reintegration into society.

## Research methods and design

### Study design

This qualitative study involved individual interviews with psychiatric inpatients admitted to Weskoppies Hospital before and during the COVID-19 pandemic. According to Leedy and Ormod ‘the qualitative approach is used to answer questions about the complex nature of phenomena, with the purpose of describing and understanding the phenomena from the participants’ point of view’.^7^ Given the study’s focus on inpatients’ subjective experiences before and during the pandemic, this design enabled a rich, in-depth understanding of how participants perceived and navigated the challenges brought about by the crisis.

### Study setting

The research was conducted at Weskoppies Hospital, which is a specialised psychiatric hospital situated on the western side of Tshwane, South Africa, that provides mental health treatment to inpatients and outpatients.^8^

### Study population and sampling

The study population included 15 psychiatric inpatients in different wards. Participants were purposively sampled, with every consecutive inpatient who met the inclusion criteria and was willing to participate and included during the data collection period, which took place between June and July 2023. The participants were patients whom the principal investigator had seen previously during routine consultations, as well as individuals referred by colleagues and nursing staff. Patients were reassured that declining would not impact their care, and participation was completely voluntary. A semi-structured interview guide that enabled participants to openly share their experiences was employed to increase trust. Reliability was reinforced by detailed documentation of the research process, and reflexivity was always upheld to reduce the principal investigator’s prior knowledge of the participants.

### Inclusion and exclusion criteria

We included psychiatric inpatients older than 18 years who were admitted before and during the pandemic. Eligible participants needed to demonstrate an understanding of the study information, provide informed consent and communicate effectively. Patients were excluded if they were under 18 years old, had no inpatient experience at Weskoppies Psychiatric Hospital prior to the pandemic or presented with conditions that would impair their ability to participate meaningfully, such as severe psychosis, significant cognitive impairment or inability to comprehend the concept of COVID-19.

To ensure participants were clinically sound to engage in the study, the principal investigator, who is a psychiatry registrar, her colleagues and nursing staff, used clinical judgement based on current mental status examinations and consultation notes, confirming that the individual was mentally stable and capable of participating in a research interview without risk to themselves or the quality of data collected.

### Data collection

In July 2023, the principal investigator conducted individual semi-structured interviews to explore psychiatric inpatients’ experiences of adapting during the COVID-19 pandemic. The interviews incorporated open-ended questions and were conducted in English, Afrikaans, Tshivenda, or Sepedi, depending on the participant’s language preference. Basic demographic data were collected on a data collection sheet (Table 1). The researcher’s created questions that would initiate conversations, provide structure, and allow the participants to freely elaborate and share their experiences of adapting to the COVID-19 pandemic while being inpatients at Weskoppies Hospital.

**TABLE 1:** Demographics of the psychiatric inpatients at Weskoppies Hospital who shared their experiences of adapting to the COVID-19 pandemic.

Demographics	*n*
**Age (years)**	
18–35	3
36–45	6
46–55	4
55–65	2
**Gender**	
Male	11
Female	4
**Admission duration**	
<5 years	2
5–10 years	10
10–15 years	2
>20 years	1

A total of 15 interviews were included in the study. Most interviews were concluded within 20 minutes, with field notes taken during the interviews. Each interview was audio recorded and then transcribed for further analysis. All participants provided written informed consent. No names or identifying data of the participants were recorded or used when reporting results. The interviews were conducted in a private, well-ventilated consultation room while always observing COVID-19 protocols.

### Data analysis

The interview transcriptions and field notes were analysed via thematic content analysis, in which a coding scheme was used to identify the most common or recurrent themes that emerged from the data.^9^ After 12 interviews, recurrent themes started to emerge, indicating data saturation.

### Ethical considerations

Ethical clearance to conduct this study was obtained from the University of Pretoria, Faculty of Health Sciences Research Ethics Committee (No. 200/2022). Written informed consent was obtained from each participant. Permission to conduct this study was obtained from Weskoppies Psychiatric Hospital management.

## Results

This study explored psychiatric inpatients’ experiences of adapting to the COVID-19 pandemic. Four themes, each with subthemes, were identified from the interviews (see Table 2). The themes included adapting within the hospital’s environment, staff as a source of stability and adaptation support, navigating family separation and connection and emotional resilience and adaptive responses.

**TABLE 2:** Themes of psychiatric inpatients at Weskoppies Hospital who shared their experiences of adapting to the COVID-19 pandemic.

Themes	Subthemes
Adapting within the hospital’s environment	Infrastructure and space limitations Continued access to healthcare Awareness and understanding of COVID-19
Staff as a source of stability and adaptation support	Consistent communication and daily updates Emotional reassurance Therapeutic support Bridging the gap with families
Navigating family separation and connection	Restricted communication Maintaining bonds through food and parcels Coping with grief, loss and bereavement
Emotional resilience and adaptive responses	Balancing fear and faith Adjusting to new norms

COVID-19, coronavirus disease 2019.

### Theme 1: Adapting within the hospital’s environment

#### Subtheme 1.1. Infrastructure and space limitations

Most participants knew what to expect when admitted to Weskoppies Hospital before the COVID-19 pandemic. They understood that mental illness was treated at the facility, and patients were rehabilitated and then discharged back into the community. The layout of the hospital infrastructure was easy to tolerate because this was a temporary setting. When the COVID-19 pandemic hit, closed wards became claustrophobic for the participants:

‘Ahh I think maybe the hardest thing would have to be like having to deal with maybe following the rules of COVID-19 like social distancing cause we all used to being in a cramped environment like being 22 patients in a ward and all of that so we had to navigate and try to keep the virus at bay, those were the tough times.’ (Participant D, male, 30 years)

‘Having access to an open ward helped me.’ (Participant Q, male, 61 years)

#### Subtheme 1.2. Continued access to healthcare

Some participants appreciated the quick response they received when they were not feeling well during the COVID-19 pandemic. They had previously been looked after; however, they felt that during the COVID-19 pandemic, the hospital had placed systems that awarded them immediate medical attention. At their homes, they only had access to their nearest healthcare facility, which was not always nearby. Being in hospital gave the participants a sense of safety that they would be attended to if anything goes wrong. The participants were more worried about their family members than about themselves:

‘The fact that they did regular testing and even now when I had flu-like symptoms, they would take the samples to laboratory.’ (Participant R, female, 63 years)

‘I think just being in a place that you are safe and you know that it’s gonna benefit you in the long run I was okay with that I could keep strong and worry about my family a little. The test stations were well prepped, and everything was well oiled and functioning, very good that was impressive to see.’ (Participant D, male, 30 years)

The hospital provided personal protective equipment (PPE) and vaccinations. Some patients had better access to these resources at the hospital than at home. This contributed to their sense of safety and security. Six out of the 15 participants indicated that receiving the vaccine was comforting and relieved their fear of death:

‘They were handing out masks, they gave us masks, they informed us about health and hygiene, hand washing they really implemented that so we can be more safe.’ (Participant E, male, 27 years)

‘We got vaccines, and we were told to use masks. And social distancing.’ (Participant P, male, 24 years)

#### Subtheme 1.3. Awareness and understanding of COVID-19

During the COVID-19 pandemic, patients generally participated in awareness campaigns. Participants were made aware of the COVID-19 pandemic. The hospital used different methods of communication, from posters placed around the hospital to staff educating them about COVID-19. Awareness campaigns and the provision of psychoeducation helped the participants to adapt during the COVID-19 pandemic:

‘They posted posters on the wall. They were wearing gloves while giving food, giving the mask, they’re also influencing us to wear masks.’ (Participant G, female, 38 years)

### Theme 2: Staff as a source of stability and adaptation support

#### Subtheme 2.1. Consistent communication and daily updates

Before the COVID-19 pandemic, there was good communication between the hospital staff and the patients. This, however, intensified during the pandemic. The participants stated that they would receive regular updates from staff members, which eased their anxiety about COVID-19:

‘The information I got from the staff members, telling us at the moment we are facing this pandemic, losing a lot of people and we need to make sure that you as a patient are safe.’ (Participant E, male, 27 years)

‘The nurses were speaking to us, informed us about it. Gave us some education.’ (Participant F, male, 44 years)

#### Subtheme 2.2. Emotional reassurance

Eight out of the 15 participants appreciated the support and reassurance they received from staff members. They knew that they were cared for beyond just receiving medication:

‘Staff members reduced my stress.’ (Participant K, male, 52 years)

#### Subtheme 2.3. Therapeutic support

The hospital staff continued to provide treatment and monitor patients, despite the world’s slowdown and potential standstill as a result of the pandemic, thereby ensuring patients’ well-being and discharge back into the community.

‘It was really impressive to find out that Weskoppies was looking out for patients and trying to keep this virus at bay as much as they could, so I was impressed to see that everything that was happening was to our benefit; it was really good to see how the nurses they rallied for us during the tough time.’ (Participant D, male, 30 years)

‘I can never say that. I’d be lying, the staff is marvellous, is perfect.’ (Participant E, male, 27 years)

‘Counselling was offered for us if we needed any.’ (Participant G, female, 38 years)

Only one participant out of the 15 was unhappy about how strict the staff had become about enforcing the new rules. However, he later understood that this was a life-or-death crisis.

#### Subtheme 2.4. Bridging the gap with families

Inpatients were able to interact with their families. Participants were visited by their families, which helped. However, during the COVID-19 pandemic, restrictions were put in place, and staff served as intermediaries between participants and family members. Being allowed that link helped the participants to cope:

‘Our social workers were the ones who were fetching our parcels from the gate when our families couldn’t bring our stuff; it was real nice to see them doing that work; it was good cause we need our parcels as they keep us going.’ (Participant D, male, 30 years)

‘Staff members were really cooperative about fetching our stuff at the gate.’ (Participant E, male, 27 years)

### Theme 3: Navigating family separation and connection

#### Subtheme 3.1. Restricted communication

Participants faced challenges such as limited family contact and reduced physical contact during the COVID-19 pandemic. The absence of home-cooked meals that provided a sense of home was also a challenge:

‘We couldn’t even get visits at some stage you know if they had to bring in food it couldn’t be cooked food because we were scared that maybe it could contain the virus and something like that.’ (Participant D, male, 30 years)

‘I wasn’t able to see my family.’ (Participant M, male, 40 years)

‘The fact that visitors were not allowed.’ (Participant L, male, 38 years)

#### Subtheme 3.2. Maintaining bonds through food and parcels

Participants were connected to their families via the delivery of parcels and food. Staff assisted in delivering the parcels to them:

‘They would come here and drop the parcels at the gate; someone had to fetch the parcel at the gate.’ (Participant E, male, 27 years)

‘My mom hasn’t visited for a long time she elderly, so she was not able to visit; she rather sent me some parcels.’ (Participant R, female, 63 years)

#### Subtheme 3.3. Coping with grief, loss and bereavement

Many participants experienced a great deal of fear related to the prospect of death. Losing fellow patients or staff members was heart-breaking. Even worse, when losing friends or family members, patients were unable to attend funerals to pay their last respects. Some patients coped by speaking to psychologists, while others expressed a strong desire to return home to find closure and honour their loved ones. Grieving in a confined hospital setting posed unique emotional challenges, but participants found ways to cope with the grief and adapt under difficult circumstances:

‘Some were my friends, and about ten people died.’ (Participant M, male, 40 years)

‘My grandfather and my uncle’s wife died.’ (Participant G, female, 38 years)

‘[… *H*]is mother. She used to come visit him, and then I was sitting with him when the mom was visiting. The mom was the one that died of COVID-19.’ (Participant I, male, 50 years)

‘My grandmother passed away, I feel so bad, I didn’t go to the burial.’ (Participant N, male, 40 years)

‘I thought [*I*] was coping with [*it*]. The only time I needed counselling, I saw a psychologist; she spoke to me about my mother’s death.’ (Participant I, male, 71 years)

### Theme 4: Emotional resilience and adaptive responses

#### Subtheme 4.1. Balancing fear and faith

Coronavirus disease 2019 was associated with fear of death, loss of loved ones, and the unknown, making coping difficult for participants. Nine out of 15 participants felt afraid of dying, but their faith in God helped them cope. They turned to prayer, read the Bible and cooperated with priests and prayer sessions:

‘We all don’t know when we will be dying, and the bible says we must not be bothered by death because I knew that the people who died were going to be in a better place.’ (Participant M, male, 40 years)

‘I was scared and thinking that I will be next, but I would pray.’ (Participant Q, male, 61 years)

‘People were dying, and I was afraid.’ (Participant L, male, 38 years)

‘Certain people being positive and negative it was very concerning because I used to watch the news and every day on Morning Live people were passing away and it was alarming.’ (Participant R, female, 63 years)

‘Praying, reading the Bible together, with other roommates and the pastors used to go ward to ward holding ceremonies.’ (Participant G, female, 38 years)

‘You have to protect yourself, knowing that people were dying made me feel so bad and I prayed.’ (Participant N, male, 40 years)

#### Subtheme 4.2. Adjusting to new norms

The participants struggled to adjust to the new norm. They had to adjust to social distancing, wear masks, wash and sanitise their hands all the time and stop smoking. Participants were consoled, knowing that these precautions would help keep the virus at bay. One patient found isolation claustrophobic, while another took it as a time for self-reflection. Three of the 15 participants were frustrated that the one thing that helps them relax (smoking) was taken away, and some were happy that they finally kicked the bad habit of smoking:

‘Ahh I think maybe the hardest thing would have to be like having to deal with maybe follow the rules of COVID-19 like social distancing.’ (Participant D, male, 30 years)

‘I was alone Yeah. I was alone in the cell. Because it’s locked in most cases, it’s locked. I felt claustrophobic.’ (Participant G, female, 38 years)

‘They stopped smoking of cigarettes because as we share cigarettes … more or less [*were*] exposed to the bacteria. It’s a coping mechanism; it relaxes your nervous system … you turn to relax and don’t think about anything serious that may affect you that time.’ (Participant E, male, 27 years)

‘Yes, it helped me because I stopped smoking for good because of the protocol that says no smoking at the hospital.’ (Participant K, male, 52 years)

## Discussion

This study explored psychiatric inpatients’ experiences of adapting during the COVID-19 pandemic at Weskoppies Hospital in South Africa. The findings revealed that while the pandemic posed multiple challenges – including fear of infection, separation from family and loss of loved ones – many patients demonstrated emotional resilience and adapted through support from hospital staff, continued access to care and personal coping mechanisms.

Importantly, participants valued the consistency of psychiatric treatment during a time when outpatient services were limited or disrupted for others. The psychological impact of grief and loss was particularly profound, yet the ability to access psychotherapy and maintain hope played a crucial role in adaptation. These insights contribute to a deeper understanding of the inpatient experience during public health crises and underscore the importance of structured support systems in facilitating adjustment and emotional recovery.

A related study conducted in Qatar explored psychiatric inpatients’ views of their mental health and the social changes they experienced during the COVID-19 pandemic. Similar to the findings at Weskoppies Psychiatric Hospital, patients in the Qatar study reported heightened anxiety, disruption of routines, and challenges related to isolation. However, while the study in Qatar focused more broadly on patients’ perceptions of social change and mental health status, the current study offers a focused exploration of how inpatients actively adapted and coped with the evolving circumstances of the pandemic. By emphasising adaptation strategies, emotional resilience and the support systems within a South African psychiatric hospital context, this study provides a unique contribution to the literature, particularly from a low- and middle-income country perspective where healthcare resources and sociocultural dynamics may differ significantly.^10^

According to Carver et al., there are two general ways for coping: engagement and disengagement.^11^ Actively handling the problem- and emotion-focused elements of stressful transactions, including problem-solving, cognitive restructuring, expressing emotions and looking for social support, is known as engagement. The following coping mechanisms are part of disengagement: self-criticism, wishful thinking and problem avoidance. People are likely to become disengaged from environmental and personal interactions because of these coping mechanisms. Wishful thinking and fantasies deflect attention from the stressor, thoughts about the situation are avoided and activities that could resolve the issue are not started through disengagement.^11^ In our study, the participants mostly used the engagement coping strategy. They actively managed stressful times by cooperating with the new rules, expressing their emotions to staff and one another and accepting social support.

People need affiliation and relatedness, enjoy socialising and value the physical presence of others. The COVID-19 directives of social distancing and isolation heightened the natural need for affiliation. Consequently, people experience loneliness.^12^ In our study, participants expressed that a lack of family contact made coping difficult during the COVID-19 pandemic.

In addition to not being able to see their families, participants had to deal with loss and were deprived of opportunities to grieve appropriately. The COVID-19 pandemic affected the way many bereaved individuals were able to cope. When people’s grief is not openly acknowledged, publicly mourned or socially supported, it is referred to as disenfranchised grief.^13^ Our participants experienced such disenfranchised grief. Fortunately, they had access to staff willing to assist them during this difficult time.

While the existing literature has highlighted the role of spirituality and religious practices as protective factors during the COVID-19 pandemic, the studies identified have not focused on psychiatric inpatients.^14,15^ These findings offer a meaningful contribution to understanding how spiritual coping mechanisms support psychiatric inpatients, a group often navigating complex emotional, cognitive and social challenges. Despite the structured and sometimes restrictive nature of the inpatient setting, participants in this study actively relied on their faith and spiritual beliefs to manage uncertainty, loss and fear. This highlights the value of recognising and supporting patients’ spiritual needs as part of comprehensive mental healthcare, particularly in the context of large-scale crises such as the COVID-19 pandemic.

Our study showed that psychiatric inpatients at Weskoppies Hospital in South Africa had multifaceted experiences while adapting during the COVID-19 pandemic. These experiences were shaped by hospital-related factors, the role and support of healthcare staff, family dynamics and personal strategies for adjustment and coping. The findings underscore the importance of addressing the diverse influences on patient well-being during crises. This can guide mental healthcare practitioners in delivering more holistic care that supports rehabilitation and reintegration, reinforcing the need for a biopsychosocial approach in inpatient psychiatric settings.

### Limitations

The limitations include the small sample size because of population specificity. The results may not represent other areas because they were obtained from a single regional hospital. To assess any trends, further data from different institutions are needed. Because this is a qualitative study, the findings lack statistical validity and objectivity. Because of our retrospective study design, using semi-structured interviews may have led to recall bias.

### Recommendations

Our study focused on psychiatric inpatients’ experiences of adapting to the COVID-19 pandemic. The national state of disaster was lifted on 05 April 2022.^2^ When dealing with the aftermath of COVID-19, we recommend that further studies investigate how COVID-19 affected psychiatric inpatients and whether this has affected their coping strategies.

## Conclusion

Psychiatric inpatients at Weskoppies Hospital adapted to the COVID-19 pandemic through support from staff, family, hospital systems and personal coping strategies. These findings highlight the need for holistic, patient-centred care that includes psychosocial and spiritual support during times of crisis.
